# Time Is Brain: Acute Control of Repetitive Seizures and Status Epilepticus Using Alternative Routes of Administration of Benzodiazepines

**DOI:** 10.3390/jcm10081754

**Published:** 2021-04-17

**Authors:** Sulaiman Almohaish, Melissa Sandler, Gretchen M. Brophy

**Affiliations:** 1 Department of Pharmacotherapy and Outcomes Science, School of Pharmacy, Virginia Commonwealth University, Richmond, VA 23298, USA; Almohaishsm@vcu.edu (S.A.); sandlerm@vcu.edu (M.S.); 2 College of Clinical Pharmacy, King Faisal University, Al-Ahsa 3198, Saudi Arabia; 3 Department of Physical Medicine and Rehabilitation, School of Medicine, Virginia Commonwealth University, Richmond, VA 23298, USA

**Keywords:** seizure, epilepsy, benzodiazepines, routes of administration, status epilepticus, repetitive seizures, cluster seizures, acute repetitive seizure

## Abstract

Time plays a major role in seizure evaluation and treatment. Acute repetitive seizures and status epilepticus are medical emergencies that require immediate assessment and treatment for optimal therapeutic response. Benzodiazepines are considered the first-line agent for rapid seizure control. Thus, various routes of administration of benzodiazepines have been studied to facilitate a quick, effective, and easy therapy administration. Choosing the right agent may vary based on the drug and route properties, patient’s environment, caregiver’s skills, and drug accessibility. The pharmacokinetic and pharmacodynamic aspects of benzodiazepines are essential in the decision-making process. Ultimately, agents and routes that give the highest bioavailability, fastest absorption, and a modest duration are preferred. In the outpatient setting, intranasal and buccal routes appear to be equally effective and more rapidly administered than rectal diazepam. On the other hand, in the inpatient setting, if available, the IV route is ideal for benzodiazepine administration to avoid any potential absorption delay. In this article, we will provide an overview and comparison of the various routes of benzodiazepine administration for acute control of repetitive seizures and status epilepticus.

## 1. Introduction

The extent of neurological damage and systemic complications caused by seizures varies based on the duration and frequency of seizures and time to seizure control [1,2,3,4,5]. Healthcare providers and caregivers should promptly recognize and treat acute repetitive seizures and status epilepticus to lower the associated morbidity and mortality [1,2,3,4,5,6]. Early treatment of acute repetitive seizures, which can lead to status epilepticus, with benzodiazepines is associated with fewer required antiseizure medications upon hospital admission and a shorter seizure duration [7,8]. This emphasizes the importance of readily available agents for rapid seizure termination that can be utilized in both inpatient and outpatient settings.

The goal of seizure treatment is to halt clinical and electrographic seizure activity in the shortest amount of time possible. Different benzodiazepine routes of administration have been studied to determine which route is the fastest and most practical without sacrificing efficacy. Although most emergent seizure treatment strategies have similar clinical efficacy in terms of seizure termination, logistical issues and practicality make some agents more appealing than others. In the case of status epilepticus (SE), after first-line benzodiazepines are administered, other therapies may be required to control seizures [9,10,11].

The intravenous (IV) route for medication administration is preferred in patients with an established IV line, but this route is not always achievable, especially in an emergent setting. To overcome this complication, other routes of administration must be considered. The intramuscular (IM) route of administration has a slower onset of action and requires advanced training, but it is a practical and efficacious alternative to the IV route [12,13]. Rectal (PR) administration of drugs permits direct absorption into the bloodstream, leading to a rapid effect without first-pass metabolism [14]. Despite this, rectal administration is typically uncomfortable for patients and the time spent positioning the patient and removing their clothes may delay administration [15,16].

Considering the obstacles associated with these routes of administration, intranasal (IN) and buccal medications have become attractive options, especially in the outpatient setting. Limitations for the IN route include small volume requirements, which may be challenging to accurately measure, and reduced bioavailability due to nasal drainage or congenital abnormalities [17]. The buccal route may be challenging to administer to patients with convulsive seizures due to the risk of finger biting and aspiration.

The pharmacokinetic and pharmacodynamic profiles of benzodiazepines utilized for the treatment of seizures must also be considered. The characteristics of the ideal benzodiazepine are fast onset, an extended duration of action allowing for urgent antiseizure medications to be administered, and no active metabolites. Therefore, the focus of this article is to summarize and highlight benzodiazepine administration strategies and pharmacotherapeutic pearls for the treatment of seizures in prehospital and inpatient settings.

## 2. Benzodiazepine Routes of Administration and Pharmacotherapeutic Considerations

Lorazepam (manufactured by Baxter Healthcare Corporation, Deerfield, IL, USA), diazepam (manufactured by Pfizer Inc., New York, NY, USA. Distributed by Hospira Inc., Lake Forest, IL, USA), and midazolam (manufactured by Pfizer Inc., New York, NY, USA. Distributed by Hospira Inc., Lake Forest, IL, USA) have been widely studied for seizure control. Healthcare clinicians should familiarize themselves with the pharmaceutical differences between these drugs, which will allow them to choose the most appropriate agent for each patient in different scenarios. Knowing which dosage forms are commercially available and which of these agents have a Food and Drug Administration (FDA) approved indication for the type of seizure being treated is also helpful for drug selection (Table 1).

The IV route of lorazepam is the most widely used for seizure control in the inpatient setting. The IM onset of action is long and its absorption is unpredictable, and the sublingual (SL) tablet is not ideal in moderate to severe cases (Table 1). Diazepam IM administration shares the same absorption issues as IM lorazepam. The IM form of midazolam is absorbed more rapidly and predictably than IM lorazepam or diazepam and, therefore, is recommended in current guidelines [9,10,31] (Table 2).

Important pharmacologic characteristics of benzodiazepines for the treatment of seizures and SE are their ability to achieve high central nervous system (CNS) concentrations and their CNS half-life. Diazepam and midazolam cross the blood–brain barrier (BBB) faster than lorazepam, but lorazepam has a longer duration of action in the CNS, resulting in prolonged seizure control (Table 3). These differences may not seem significant, but the additional time allotted to initiate urgent antiseizure medications is beneficial.

If one benzodiazepine is not available due to limited resources or shortages, comparable doses of another agent should be initiated. Unfortunately, there is limited evidence supporting benzodiazepine dose conversion strategies in patients with seizures, and conversion estimates are usually based on expert opinion and clinical practice.

## 3. Pharmacokinetic and Pharmacodynamic Properties

All benzodiazepines are γ-aminobutyric acid-A (GABA_A_) agonists, but differences in their pharmacokinetic profile make each one unique. When considering the use of benzodiazepines for seizure control, it is important to understand each drug’s pharmacokinetic characteristics, including absorption, distribution, metabolism, and excretion, which affect the timing and duration of effect of these agents. Intravenous drugs are 100% bioavailable and have the quickest onset of action, followed by intranasal, rectal, IM, and SL routes of administration for most benzodiazepines. Buccal midazolam (i.e., Buccolam^®^, Epistatus^®^) has a similar onset of action to IM midazolam, but its bioavailability is significantly lower than the IM formulation [32,33,34]. As the buccal formation is not available in the United States, some clinicians use the injectable midazolam solution buccally, which has a comparable bioavailability and onset of action to Buccolam^®^ and Epistatus^®^ [32,33,34]. Buccal diazepam is under investigation, in phase 3 clinical trials [52].

Regarding the duration of action, both serum and CNS half-lives need to be considered in patients with seizures. The serum half-life of diazepam is longer than that of lorazepam, midazolam and clobazam, but both midazolam and diazepam have short CNS half-lives due to their higher lipophilicity. Lorazepam and clobazam have lower lipophilicity, and lorazepam has an onset of action of 3–5 min as compared to diazepam and midazolam, which cross the blood–brain barrier faster (e.g., 1–2 min) [47,50,51]. Due to this high lipophilicity, diazepam and midazolam also quickly redistribute out of the brain and only allow for short (e.g., <30 min) durations of seizure control when given intermittently. Therefore, lorazepam is the preferred IV benzodiazepine for acute seizure control. For patients diagnosed with status epilepticus, drugs with a short CNS duration of action are desirable for administration as a continuous infusion, allowing for rapid titration and neurological assessments. Table 2 and Table 3 summarize pharmacokinetic and pharmacodynamic characteristics of benzodiazepines in the serum and CNS, respectively.

Renal and hepatic function should also be considered, especially if a benzodiazepine is to be given as a continuous IV infusion or frequently enough that accumulation may occur. Diazepam, midazolam, and clobazam are metabolized by the liver via CYP3A4 to their active metabolites, so caution should be used in patients with hepatic dysfunction as their duration of action may be prolonged [53]. Drug–drug interactions should also be considered as agents such as voriconazole, fluconazole, and amiodarone will inhibit the activity of CYP3A4 while primidone, phenytoin, phenobarbital, and rifampin will induce its activity [53]. In patients with renal dysfunction, the active metabolite of midazolam may accumulate over time, so both hepatic and renal function should be considered if using this agent as a continuous infusion over an extended duration of time.

## 4. Administration Route Comparisons

When considering which route of administration is best, the benefits and limitations of each route should be considered. The intravenous route of administration provides the fastest onset of action with 100% bioavailability, but its application requires higher training skills and a longer time to achieve compared to other routes. Both lorazepam and diazepam IV formulations contain propylene glycol, which may cause serious side effects such as metabolic acidosis and renal insufficiency and is incompatible with most IV fluids. Lorazepam 2 mg/mL solution for injection contains 80% propylene glycol and diazepam 5 mg/mL solution for injection contains 40% propylene glycol [54]. This becomes a more important consideration when these drugs are given as a continuous IV infusion or are frequently administered. Patients with renal dysfunction are more susceptible to the accumulation of propylene glycol and its toxic effects.

Intramuscular injection absorption might be altered based on the needle size and the weight of the patient while buccal routes provide rapid absorption. However, the buccal route of administration may be challenging in patients with convulsive seizures. Rapid absorption and a large amount of drug can be administered rectally but it may cause local irritation and is inconvenient for adult patients. The intranasal route is a more attractive option than rectal in the adolescent and adult patient for these reasons. The advantages and disadvantages of each route of administration are described in Table 4.

To overcome some of the challenges of IV, IM, and PR routes of administration, benzodiazepine IN sprays were developed. Nayzilam^®^ (IN midazolam) and Valtoco^®^ (IN diazepam) are the most recently approved benzodiazepine products. These products have similar administration instructions and costs, but Nayzilam^®^ might be preferred in patients with a higher seizure frequency as the second dose may be administered after 10 min and used for one seizure cluster every 3 days [26]. The second dose of Valtoco^®^ may be applied after 4 h, but should not be used for another seizure cluster until 5 days later [22].

## 5. Evidence for Routes of Administration

To determine if various routes of administration are equally effective, several studies have been conducted. With rectal diazepam being the most studied route of administration in the outpatient setting and intravenous benzodiazepines being the drug of choice in the inpatient setting, many of these studies have used rectal and IV administrations as a comparator group for alternative routes. The evidence for alternative routes of administration is summarized in Table 5 and provides guidance in the decision-making process for identifying safe, effective, and convenient benzodiazepine routes of administration for patients in both the in- and outpatient settings.

The landmark study evaluating benzodiazepine routes of administration for adult and pediatric patients with status epilepticus is the RAMPART trial [12]. This study demonstrated noninferiority of IM midazolam and IV lorazepam for status epilepticus, although time from drug administration to seizure cessation is slower with IM midazolam than IV lorazepam. IM midazolam has a better pharmacokinetic profile as compared to IM lorazepam and IM diazepam, and it does not contain propylene glycol in its formulation. The weight of the patient should be considered when dosing IM midazolam as patients with a higher body mass index have a higher rate of failing IM treatment [79]. None of the studies on routes of IM administration to date have compared patients based on weight and this might contribute to the success and failure of IM therapy. Overall, IM and IV benzodiazepines appear to be equally effective in controlling seizures, but the IV route works faster and is therefore the preferred route of administration in the acute setting for trained personnel. In cases of drug shortages or for patients without IV access, IM midazolam is definitely a viable option for the treatment of acute seizures and status epilepticus.

Studies of intranasal benzodiazepines in the past have utilized a non-FDA approved product, the intravenous injectable solution administered with an atomizer, compared to the newer commercially available IN products. No studies to date have compared the newest IN products with the off-label use of the IV solution administered intranasally. Older studies using the IV diazepam solution intranasally have shown it to be effective and that it sometimes more rapidly terminates seizures than IV and rectal diazepam, possibly due to faster administration times [76]. In a meta-analysis, IN midazolam showed no significant difference in seizure cessation within 10 min when it was compared to IV diazepam [65]. However, the time to seizure control after the presentation to the emergency department was shorter in the IN midazolam group. It is recommended that the volume for intranasal drug not exceed 0.2 mL [59]; however, most of the studies evaluated exceeded that limit. Intranasal diazepam has higher bioavailability compared to the midazolam injectable solution given intranasally, which may also affect the efficacy of the total dose if not completely absorbed. Despite these limitations, the intranasal route of administration demonstrates a similar efficacy to intravenous benzodiazepine therapy in seizure control.

There is limited evidence that compares buccal benzodiazepine administration to IV for seizure control, with most of these studies being conducted in the pediatric population. Some countries have buccal midazolam products available on the market while others use the intravenous injectable solution as an alternative for administration via this route [65]. Buccal midazolam and diazepam are effective for acute seizure treatment and have demonstrated similar efficacy to IV diazepam [77,78]. It is important to note that some patients in these studies received commercially available buccal midazolam (Epistatus^®^) while others received injectable midazolam buccally, which may have impacted the results. Buccal diazepam has less variability in achieving maximum concentrations compared to rectal diazepam, but the time to reach maximum concentrations is longer [69,80]. One important factor to consider is that food, saliva secretion, and local pH may alter the absorption of the buccal films and it may reduce their maximum concentration [60]. Buccal diazepam is currently in phase 3 investigational drug trials in the United States [52].

Studies comparing oral routes of administration to control emergent seizure attacks have also been conducted; although these routes are usually prescribed for patients with panic attacks. In regard to efficacy and convenience, patients with a history of rectal diazepam use reported that they felt oral clonazepam was equally or more effective than rectal diazepam, and caregivers preferred this route over rectal as well [70]. For pediatric convulsive status epilepticus, sublingual lorazepam was found to be less effective in stopping seizures within 10 min of administration compared to rectal diazepam, and the odds of treatment failure was higher in the lorazepam group [73]. They also observed a high potential for failure for oral therapies in patients with convulsive seizures due to the risk of aspiration or incomplete absorption due to salivation [73]. Although oral routes of administration are an option to manage patients with seizure attacks; in patients presenting with convulsive seizures, this route is not practical.

## 6. Benzodiazepine Routes and Adverse Events

Several adverse events are associated with benzodiazepine administration, including hypotension, confusion, sedation, and respiratory failure. However, a delay in benzodiazepines administration lowers the potency of benzodiazepines due to the changes in receptors, including GABA downregulation and *N*-methyl-d-aspartate (NMDA) upregulation, after a prolonged seizure [81,82]. A multicenter prospective clinical trial of pediatric patients found that a delay of benzodiazepine administration for more than 10 min resulted in higher chances of requiring continuous infusion therapy (adjusted odds ratio (AOR), 1.8; 95% CI, 1.01–3.36; *p* = 0.047), longer convulsion duration (AOR, 2.6; 95% CI, 1.38–4.88; *p* = 0.003), more frequent hypotension (AOR 2.3; 95% CI, 1.16–4.63; *p* = 0.02), and death (AOR, 11.0; 95% CI, 1.43 to ∞; *p* = 0.02) [83]. In adults, 22.5% of patients who received placebo therapy experienced either hypotension, cardiac dysrhythmia, or a respiratory problem that required an intervention compared to 10.6% in the IV lorazepam group and 10.3% in the IV diazepam group [7].

Chronic or continuous exposure to benzodiazepines may increase the risk of developing tolerance leading to decreased efficacy [63,64]. This is concerning in patients receiving continuous infusion benzodiazepines for status epilepticus, and will require increasing doses for continued seizure control, but it may not occur with intermittent doses of benzodiazepines to control acute repetitive seizure episodes [9,10,84]. Once maximum doses of the selected benzodiazepine have been reached, other antiseizure drugs may need to be initiated to control seizures [9,10].

In summary, a delay in the administration of a benzodiazepine agent will decrease the ability to control a seizure and may lead to death. The risk of withholding a benzodiazepine from a patient with a seizure far outweighs the adverse effects. Some of the benzodiazepine adverse events associated with the various routes of administration are summarized in Table 6.

## 7. Summary

In the outpatient setting, treatment of acute repetitive seizures was previously limited to rectal diazepam, whereas in the inpatient setting IV benzodiazepines remain the route of choice for seizure emergencies. New studies have compared rectal diazepam to IN midazolam, IM midazolam, buccal midazolam, IM diazepam, buccal diazepam, sublingual lorazepam, and an orally disintegrating clonazepam tablet. The limitations of these trials include the use of intravenous injectable solutions for IN and buccal administration and small sample sizes. Intranasal and buccal routes appear to be equally effective and more rapidly administered than rectal diazepam. The choice of the route of administration in the outpatient setting should consider the logistical challenges of administration, patient and caretaker comfort, and cost.

In the inpatient setting, the IV route remains the preferred route for benzodiazepine administration, and lorazepam is the preferred agent due to its longer CNS half-life. Alternative routes of administration for benzodiazepines include IM, IN, and buccal administration and these may be used in patients without IV access. All routes of benzodiazepine administration are effective for acute seizure management, but pharmacokinetic characteristics, patient comorbidities, and concomitant medications should also be considered in the inpatient setting when choosing the specific agent and when multiple doses are required.

Benzodiazepines may cause adverse events which vary in frequency depending on the route of administration. Despite this, a delay or inappropriate dosing of benzodiazepine therapy to control seizures can be detrimental and may lead to poor outcomes.

## Figures and Tables

**Table 1:** Benzodiazepine products, routes, FDA indications, and cost [18,19,20,21,22,23,24,25,26,27,28,29,30].

Agent	Routes of Administration	FDA Indications	Other Considerations	Cost (AWP)
Lorazepam	(IV, IM, IN, SL, Oral solution)	IV approved for status epilepticus Other routes used off-label for acute active seizure	IV solution contains propylene glycol IM onset of action is variable [14] SL not available in the US	Ativan^®^—IV/IM: USD 2.28/2 mg vial Lorazepam—IV/IM: USD 0.60–4/2 mg vial Ativan Intensol^®^—Oral solution: USD 1.60/2 mg
Diazepam	(IV, IM, PR, IN)	IV approved for status epilepticus IV and rectal approved for acute seizure activity IN approved for acute repetitive seizures IM autoinjector approved for the US military use; however, not commercially available	IV solution contains propylene glycol IM onset of action variable [14]	Diazepam—IV/IM: USD 11–16/5 mg Diastat^®^—PR: USD 421/10 mg Valtoco^®^—IN: USD 336/5 mg
Midazolam	(IV, IM, buccal, IN)	IN approved for acute repetitive seizures Other routes used off-label for status epilepticus or other seizure types	Buccal not commercially available in the US	Midazolam—IV/IM: USD 3.43/5 mg Nayzilam^®^—IN: USD 330/5 mg
Clobazam	(Oral: tablet, film, susp.)	Approved for Lennox-Gastaut as an adjunctive therapy Other indications used off-label for catamenial epilepsy, refractory seizures, and recurrent seizures	All oral forms are available in the US	Sympazan^®^—oral film: USD 17.22/5 mg Onfi^®^—oral suspension: USD 12.67/2.5 mg

AWP—Average wholesale price in the United States. IM, intramuscular; IN, intranasal; IV, intravenous; PR, rectal; Susp, suspension. Ativan^®^ manufactured by MEDA Manufacturing GmbH Cologne, Germany for Valeant Pharmaceuticals North America LLC, Bridgewater, NJ, USA. Ativan Intensol^®^ marketed by Paddock Laboratories Inc., Minneapolis, MN, USA. Lorazepam manufactured by Baxter Healthcare Corporation, Deerfield, IL, USA. Diazepam manufactured by Pfizer Inc., New York, NY, USA and distributed by Hospira Inc., Lake Forest, IL, USA. Diastat^®^ manufactured by DPT Laboratories Ltd, San Antonio, TX, USA for Valeant Pharmaceuticals North America LLC, Bridgewater, NJ, USA. Valtoco^®^ manufactured by Catalent Pharma Solutions, Morrisville, NC, USA. Midazolam manufactured by Pfizer Inc., New York, NY, USA and distributed by Hospira Inc., Lake Forest, IL, USA. Nayzilam^®^ manufactured for Proximagen, LLC, Plymouth, MN, USA. Sympazan^®^ manufactured by Aquestive Therapeutics Warren, NJ, USA. Onfi^®^ manufactured by Rosemont Pharmaceuticals, Ltd. Leeds, West Yorkshire, U.K for Lundbeck, Deerfield, IL, USA.

**Table 2:** Benzodiazepine pharmacokinetic and pharmacodynamic characteristics by route of administration [14,18,19,20,22,23,24,25,26,28,29,30,32,33,34,35,36,37,38,39,40,41,42,43,44,45,46].

Agent	Dose (maximum)	Route	Onset (min)	Duration of Action	Bioavailability (%)	Volume of Distribution (L/kg)	Metabolism	Excretion	T_1/2_ (h)
Lorazepam (Ativan^®^ Injection)	0.1 mg/kg (4 mg)	IV	1.6	4–6 h	100	1.3	Liver to inactive metabolites	Hepatic metabolism to inactive metabolites	12
	-	IM	12–19		83–100				
Lorazepam (Ativan Intensol^®^)	0.1 mg/kg (4 mg)	SL	Readily	-	90				
Lorazepam (Ativan^®^ Sublingual tablets) *	0.1 mg/kg (4 mg)	SL	15–17	-	>90				
Midazolam (Versed^®^)	0.2 mg/kg (10 mg)	IV	1.5–2.5	30–80 min	100	1–3	CYP3A4 to active metabolite	Renal	3–4
Midazolam (Versed^®^)	0.2 mg/kg (10 mg)	IM	5–15	2–6 h	>90				3–5
Midazolam (Nayzilam^®^)	0.2 mg/kg (15 mg)	IN	3–10	23 min	44				2–6
Midazolam	0.2 mg/kg (15 mg)	IV injection given IN	6–14	-	44–83				2–4
Midazolam (Buccolam^®^, Epistatus^®^) *	0.5 mg/kg (30 mg)	Buccal	5–15	-	75–87				3–4
Midazolam (Versed^®^)	0.5 mg/kg (30 mg)	IV injection given Buccal	~15	-	75				-
Diazepam (Valium^®^)	0.15 mg/kg (10 mg)	IV	1–3	15–30 min	100	0.8–1.2	CYP2C19 and CYP3A4 to active metabolites	Renal	33–45
Diazepam (Valium^®^)	-	IM	~15	-	>90				60–72
Diazepam (Valtoco^®^)	0.2 mg/kg (20 mg)	IN	2–10	15–30 min	97				~49
Diazepam	0.2 mg/kg (20 mg)	IV injection given IN	1–10	-	Up to 74				17–33
Diazepam (Diastat^®^)	0.2 mg/kg (20 mg)	PR	2–10	15–30 min	90				~46
Clobazam (Onfi^®^)	0.2 mg/kg (40 mg)	Oral Susp	19–30	-	87	100	CYP2C19 and CYP3A4 to active metabolites	Renal	16–82
Clobazam (Sympazan^®^)	0.2 mg/kg (40 mg)	Oral Film							

* Not available in the US; IV injectable solution is used for buccal administration in the US; IM, intramuscular; IN, intranasal; IV, intravenous; PR, rectal; Susp, suspension. Ativan^®^ sublingual tablet manufactured by Pfizer Inc., Kirkland, Quebec, Canada. Buccolam^®^ Shire Pharmaceuticals Ireland Limited, Dublin, Ireland. Veriton Pharma, Weybridge, Surrey, UK.

**Table 3:** Central nervous system pharmacokinetic characteristics of benzodiazepines [47,48,49,50,51].

CNS Characteristics	Lorazepam	Diazepam	Midazolam	Clobazam
Lipophilicity	Low	High	High	Low
CNS half-life	Long	Short	Short	Long
CNS penetration	Slow	Fast	Fast	Slow
CNS concentration	5–15% of serum	3–12% of serum	3–14% of serum	30–34% of serum
Onset of EEG slow-wave, minutes	3.8 ± 3.1	0.89 ± 3.1	0.29 ± 0.04	0.44 ± 0.16
Duration of EEG slow-wave, minutes	28.3 ± 10.1	7.5 ± 1.4	6.3 ± 1.9	62.7 ± 13.9

CNS, central nervous system; EEG, electroencephalogram.

**Table 4:** Administration route advantages and disadvantages [55,56,57,58,59,60,61,62,63,64].

Route	Advantages	Disadvantages
Intravenous	Fastest onset of action No bioavailability concerns Bypass first-pass metabolism Large volume can be administered Reliable route for sedated or comatose patients	Highly trained individuals required to administer May require physical restraint Injection site irritation, extravasation, or infection Development of tolerance with continuous infusions
Intramuscular	Bypass first-pass metabolism Prolonged action	Trained individuals required to administer May require physical restraint Painful Variable absorption Injection site irritation or infection Limited volume can be administered (2–5 mL)
Intranasal	Easily administered Rapid absorption Bypass first-pass metabolism Direct drug delivery to blood-brain-barrier	Small volume (≤0.2 mL) hard to measure Short retention time Nasal discharge may alter the absorption Nasal irritation
Buccal and Sublingual	Rapid absorption Bypass first-pass metabolism Low risk of infection	Variable absorption Unpalatable Challenging to place and maintain in the correct location Risk of aspiration
Rectal	Bypass first-pass metabolism Ease of administration in infant emergencies Low risk of infection	Rectal irritation or proctitis with ulceration Less convenient for adults

**Table 5:** Evidence supporting alternative routes of benzodiazepines administration.

**Intranasal and Rectal Formulation Trials**					
**Author/Year**	**Design (Type of Seizure)**	**Setting**	**Sample**	**Interventions**	**Results**
Jain et al. 2016 [65]	Systematic review and meta-analysis (no specific type of seizure)	Emergency department	Adult and pediatric; two randomized open-label trials (N = 91)	IN midazolam (0.2 mg/kg) Rectal diazepam (0.3–0.5 mg/kg)	IN midazolam is superior to rectal diazepam in terminating seizures within 10 min (RR 1.14; 95% CI, 1.05–1.25)
Holsti et al. 2010 [16]	Randomized single-blind trial (no specific type ofseizure)	Outpatient	Pediatrics (N = 92)	IN midazolam (0.2 mg/kg) Rectal diazepam (0.3–0.5 mg/kg)	Time to seizure cessation after drug administration was not significantly different between IN midazolam and rectal diazepam (3 vs. 4.3 min, *p* = 0.09)
Haan et al. 2010 [66]	Cross-sectional (no specific type ofseizure)	Residential epilepsy center	Adults (N = 21)	IN midazolam (10 mg) Rectal diazepam (10 mg)	Both have similar success rate (82% vs. 89%, *p* = 0.57) and time to seizure termination (4.6 vs. 4.3 min, *p* = 0.6)
**Intramuscular and Rectal Formulation Trials**					
**Author**	**Design (Type of Seizure)**	**Settings**	**Sample**	**Doses**	**Results**
Momen et al. 2015 [67]	Randomized open-label trial (status epilepticus)	Emergency department	Pediatrics (N = 100)	IM midazolam (0.3 mg/kg) Rectal diazepam (0.5 mg/kg)	Both have a similar success rate in seizure control, but midazolam is faster to achieve seizure cessation after drug administration (2.17 vs. 1.1 min, *p* < 0.001)
Lamson et al. 2011 [39]	Pharmacokinetic study (healthy subjects)	-	Adults (N = 48)	-	Similar bioavailability, but rectal diazepam had a faster time to maximum concentration (0.17–1.00 h vs. 0.25–2.00 h)
**Buccal and Rectal Formulation Trials**					
**Author**	**Design (Type of Seizure)**	**Settings**	**Sample**	**Doses**	**Results**
Jain et al. 2016 [65]	Systematic review and meta-analysis (no specific type of seizure)	Mostly in the emergency department	Pediatric and adult patients of 7 randomized clinical trials	Buccal midazolam (0.25–0.5 mg/kg) Rectal diazepam (0.5 mg/kg)	Buccal midazolam has a significantly higher rate of seizure cessation within 10 min compared to rectal diazepam (RR 1.14; 95% CI, 1.06–1.24; *p* = 0.0008)
Nakken and Lossius, 2011 [68]	Quasi-randomized trial (status epilepticus)	Residential institution	Adults (N = 22)	Buccal midazolam (mean 15.5 mg) Rectal diazepam (mean 26 mg)	Buccal midazolam has a significantly shorter time to convulsive status epilepticus control (2.8 vs. 5 min, *p* = 0.012)
Rogawski et al. 2020 [69]	Pharmacokinetic study (no specific type of seizure)	-	Adults (N = 28)	-	Buccal diazepam is less variable in reaching the maximum concentration compared to rectal diazepam (buccal geometric SD [GSD] 136.12–306.49 and rectal GSD 87.71–508.63), but the time buccal formulation takes to reach maximum concentration is longer than the rectal formulation (1.0 and 0.52 h, *p* < 0.05)
**Other Oral Formulations Trials**					
**Author**	**Design (Type of Seizure)**	**Settings**	**Sample**	**Doses**	**Results**
Troester et al. 2010 [70]	Cross-sectional home response from caregivers (no specific type of seizure)	Outpatient	Pediatrics (N = 38)	Clonazepam oral disintegrating tablet (0.25–2 mg)	Clonazepam achieved seizure control within 10 min for all the patients (74% within 5 min), and 69% of the patients who used rectal diazepam previously felt oral clonazepam was as equal or more effective than rectal diazepam
Conry et al. 2009 [71]	Randomized, double-blind multicenter trial (drop vs. nondrop seizures, repetitive seizures were recorded)	Outpatient	Adults and pediatrics (N = 68)	Clobazam oral tablet (0.25 mg/kg/day vs. 1 mg/kg/day)	Patients received daily doses of clobazam. In both drop and nondrop seizures, high-dose significantly reduced seizure frequency compared to low-dose (*p* = 0.0001 and *p* = 0.0222, respectively)
Feely et al. 1982 [72]	Placebo-controlled cross-over (repetitive seizures and catamenial epilepsy)	Outpatient	Adolescent (N = 14)	Clonazepam, did not specify the dosage form (20–30 mg/day)	Patients received daily doses of clobazam for 10 days around menstruation time. Thirteen patients responded favorably to clobazam. Three of them have been successfully treated to be seizure free for 3–3.5 years. No evidence of tolerance was observed
Malu et al. 2014 [73]	Randomized open-label multicenter trial (status epilepticus)	Emergency department	Pediatrics (N = 436)	Sublingual lorazepam (0.1 mg/kg) Rectal diazepam (0.5 mg/kg)	Sublingual lorazepam is less effective in stopping seizures within 10 min compared to rectal diazepam (56–79%, *p* < 0.001), and the treatment failure was higher in the lorazepam group (OR = 2.95, 95% CI = 1.91–4.55)
**Intravenous and Intramuscular Trials**					
**Author**	**Design (Type of Seizure)**	**Settings**	**Sample**	**Doses**	**Results**
Welch et al. 2015 [74]	Randomized double-blind multicenter trial (status epilepticus)	Emergency department	Pediatrics (N = 120)	IM midazolam (0.5 mg/kg) IV lorazepam (0.1 mg/kg)	IM midazolam have similar efficacy to IV lorazepam in stopping seizure before emergency department arrival (68.3% in IM midazolam vs. 71.6% in lorazepam) and (risk difference = −3.3%; 99% CI −24.9% to 18.2%)
Portela et al. 2015 [75]	Randomized open-label trial (no specific type of seizure)	Emergency department	Pediatrics (N = 32)	IM midazolam (0.5 mg/kg) IV diazepam (0.5 mg/kg)	The time from admission to seizure termination was shorter in the IM midazolam group (7.3 vs. 10.6 min; *p* = 0.006), and IV placement was unsuccessful in 25% of patients in the IV group
Silbergleit et al. (RAMPART) 2012 [12]	Randomized double-blind multicenter trial (status epilepticus)	Emergency department	Adults and pediatrics (N = 893)	IM midazolam (5–10 mg) IV lorazepam (2–4 mg)	IM midazolam was noninferior to IV lorazepam with regard to the rate of seizure control without the need for rescue therapy (73.4% vs. 63.4% [95% CI, 4.0–16.1; *p* < 0.001]), and the total time to seizure cessation from randomization was similar between both groups
**Intravenous and Intranasal Trials**					
**Author**	**Design (Type of Seizure)**	**Settings**	**Sample**	**Doses**	**Results**
Inokuchi et al. 2015 [76]	Retrospective cohort study (status epilepticus)	Emergency department	Adults (N = 19)	IN diazepam (10 mg) IV diazepam (10 mg)	IN diazepam demonstrated a significantly shorter time to seizure termination than IV diazepam (3 vs. 9.5 min, *p* = 0.003)
Jain et al. 2016 [65]	Systematic review and meta-analysis (no specific type of seizure)	Emergency department	Pediatric patients of 4 randomized clinical trials	IN midazolam (0.2 mg/kg) IV diazepam (0.2–0.3 mg/kg)	IN midazolam is similar to IV diazepam in seizure cessation within 10 min (RR 1.00; 95% CI, 0.93–1.06). However, the time to seizure control after the presentation to the emergency department was shorter in the IN midazolam group (mean difference −5.23 min; 95% CI, −9.55 to −0.90)
**Intravenous and Buccal Formulation Trials**					
**Author**	**Design (Type of Seizure)**	**Settings**	**Sample**	**Doses**	**Results**
Tonekaboni et al. 2012 [77]	Randomized open-label trial (status epilepticus)	Emergency department	Pediatrics (N = 92)	Buccal midazolam (6–12 mo: 2.5 mg; 1–4 yrs: 5 mg; 5–9 yrs: 7.5 mg; ≥10 yrs: 10 mg) IV Diazepam (0.3 mg/kg)	Both medications have similar efficacy in seizure control within 10 min (68.8% vs. 70%, *p* = 0.09)
Talukdar and Chakrabarty, 2009 [78]	Randomized open-label trial (no specific type of seizure)	Emergency department	Pediatrics (N = 120)	Buccal midazolam (0.2 mg/kg) IV diazepam (0.3 mg/kg)	Buccal midazolam and IV diazepam have similar efficacies in controlling any type of seizure within 5 min of drug administration (85% vs. 93.3%, *p* = 0.142). However, it took buccal midazolam a shorter time to control the seizure after the presentation to the emergency department compared to IV diazepam (*p* = 0.004)

**Table 6:** Benzodiazepine adverse events associated with route of administration [12,16,18,20,22,23,24,25,26,29,30,66,68,76,77,78,85,86,87,88,89,90,91,92,93,94].

Adverse Event	Lorazepam	Midazolam			Diazepam			Clobazam
	IV *	IM	IN	Buccal	IV *	IN	PR	Oral
Respiratory complication	4.4–17.6%	10.8–14.6%	8%	1.2–4.6%	5.6–16.8%	1–6%	1.2–6.4%	13–14%
Hypotension	1.5–25.8%	1.4%	<1–21.8%	<1%	15–31.6%	2%	2–4.4%	<1%
Cardiac arrhythmia	7.2%	<1%	<1%	<1%	1–2.1%	<1%	<1%	<1%
Dizziness/ataxia	7%	<1%	<1%	<1–4.6%	3–6%	6%	4.4–11%	10%
Somnolence/drowsiness	1.5%	<1%	10–68%	1–21%	23%	23–57%	7–55%	16–25%
Local reaction	<1%	1.1–5%	17–29%	<1–21%	<1%	14%	7%	<1%

* Formulations that contain propylene glycol have a higher risk of metabolic acidosis, renal insufficiency, hypotension, and cardiac arrhythmias. IM, intramuscular; IN, intranasal; IV, intravenous; PR, rectal.
